# Meeting the Challenge of Controlling Viral Immunopathology

**DOI:** 10.3390/ijms25073935

**Published:** 2024-04-01

**Authors:** Engin Berber, Sachin Mulik, Barry T. Rouse

**Affiliations:** 1 Infection Biology, Lerner Research Institute, Cleveland Clinic, Cleveland, OH 44195, USA; berbere3@ccf.org; 2 Center for Biomedical Research, The University of Texas Health Science Center at Tyler, Tyler, TX 75708, USA; dr.sachin.mulik@gmail.com; 3 College of Veterinary Medicine, University of Tennessee, Knoxville, TN 37996, USA

**Keywords:** viral infection, immunopathology, immunotherapy, immunometabolism

## Abstract

The mission of this review is to identify immune-damaging participants involved in antiviral immunoinflammatory lesions. We argue these could be targeted and their activity changed selectively by maneuvers that, at the same time, may not diminish the impact of components that help resolve lesions. Ideally, we need to identify therapeutic approaches that can reverse ongoing lesions that lack unwanted side effects and are affordable to use. By understanding the delicate balance between immune responses that cause tissue damage and those that aid in resolution, novel strategies can be developed to target detrimental immune components while preserving the beneficial ones. Some strategies involve rebalancing the participation of immune components using various approaches, such as removing or blocking proinflammatory T cell products, expanding regulatory cells, restoring lost protective cell function, using monoclonal antibodies (moAb) to counteract inhibitory molecules, and exploiting metabolic differences between inflammatory and immuno-protective responses. These strategies can help reverse ongoing viral infections. We explain various approaches, from model studies and some clinical evidence, that achieve innate and adaptive immune rebalancing, offering insights into potential applications for controlling chronic viral-induced lesions.

## 1. Introduction

Virus infections occur in all animals and take on many guises. The great majority are silent infections, and only a few exert a devastating outcome in those affected. However, residing in an infected vertebrate host is problematic for a virus unless it has strategies to bypass or manage recognition and rejection by the host’s immune reactions. With some infections, the attempts at control by the immune system are unsuccessful, and then the response itself becomes chronic, resulting in a tissue-damaging lesion that is considered to be immunopathological. Such responses were brought into prominence recently when the worldwide pandemic of SARS-CoV-2 virus infection appeared on the scene. Whereas many infections by SARS-CoV-2 were mild and short-lived and were controlled rapidly at the entrance sites by the immune system, if spread occurred to systemic locations, particularly to the lower respiratory tract, the vigorous immune response that became the major cause of tissue damage and the dire consequences that often followed [1,2]. The most effective treatments for this scenario were those that dampened the immune reactions rather than therapies directed at the virus. The lesions caused by several additional human viral infections are judged as mainly immunopathological rather than being the direct result of the virus replicating in host cells. Examples include dengue shock syndrome, that usually occurs in persons exposed to a different Dengue virus strain from their initial infection. Others include the liver lesions caused by Hepatitis B (HBV) and C (HCV) viruses, the lesions in the cornea and the central nervous system (CNS) that result from herpes simplex virus (HSV), lesions in the respiratory tract caused by Respiratory syncytial virus (RSV) and several instances where viruses infect the CNS. Table 1 lists some examples of virus infections where at least some of the lesions that occur are judged to represent immunopathological reactions to infections.

The good news with respect to viral immunopathology is that when lesions have been understood at a mechanistic level, it has become apparent that whereas some aspects of host response activity are direct mediators of tissue damage, there are other components ongoing at the same time that are counter-inflammatory and, if left alone, might resolve the lesions. This situation raises the prospect that if ways could be found to rebalance the participation of the various host immune activities, then lesions would be minimized and perhaps also the virus relinquished. Clinicians have achieved success with inhibiting inflammatory reactions and have relied mainly on using powerful drugs such as corticosteroids that inhibit several inflammatory events. Unfortunately, relying on corticosteroids is far from ideal, especially if used for prolonged periods, since several side effects can occur [3]. The mission of this review is to identify immune damaging participants involved in antiviral immunoinflammatory lesions that could be targeted and their activity changed selectively by maneuvers that may not diminish the impact of components that help resolve lesions. Ideally, we need to identify therapeutic approaches that can reverse ongoing lesions that lack unwanted side effects and are affordable to use. Hopefully, these therapies do not prove to be as elusive to find as the holy grail!

**Table 1:** Some selected viral infections where immune responses involved in tissue damage.

Disease	Immunopathogenesis	Key Immune Cells/Cytokines	Refs.
Dengue virus	Formation of immune complexes (virus-antibody) depositing in blood vessels, triggering inflammation and vascular leakage. Cytokine storm resulting from infection of inflammatory cells	B cells, defective CD4+ and CD8+ T cells, macrophages; Dengue-specific antibodies, TNF-α, IL-2, IL-6	[4,5,6]
EBV	Potential molecular mimicry triggering autoimmune reactions against self-tissues	CD4+ and CD8+ T cells, B cells; EBV-specific antibodies	[7,8]
HBV	Chronic infection triggers CD8-mediated inflammation, leading to liver damage.	B cells, CD4+ and CD8+ T cells, macrophages; HBV-specific antibodies, IFN-γ, TNF-α, IL-1, IL-6	[9]
HCV	Immune complex deposition leads to chronic inflammation and liver damage.	B cells, macrophages; HCV-specific antibodies	[10]
HSV	T cell-mediated chronic inflammatory response in eye and brain	CD4+ and CD8+ T cells, NK cells, IFN-γ, TNF-α, IL-1, IL-6, IL-17	[11,12,13]
LCMV	T cell-mediated inflammation and Immune complexes in kidney and skin	CD8+ T cells, macrophages; IFN-γ, TGF-beta, IL-10, IL-7. May also involve CD4+ T cells and B cells in specific contexts.	[14]
RSV	Th2-biased immune response with release of proinflammatory cytokines and eosinophil recruitment	Neutrophils, ROS production, Netosis, NLRP3, CD4+ T cells, eosinophils; IL-3, IL-4, IL-5, IL-10, IL-13, IL-17	[15,16]
SARS-CoV-1 and 2	Combined inflammatory response (cytokine storm) and direct viral damage to endothelial cells, and T cell-mediated damage to endothelial cells	CD4+ and CD8+ T cells, macrophages, NK cells; IL-1, IL-6, TNF-α, IFN-γ.	[17,18,19]

EBV: Epstein–Barr virus; HBV: Hepatitis B virus; HCV: Hepatitis C virus; HSV: Herpes simplex virus; IFN-γ: Interferon-gamma; NK cells: Natural killer cells; NLRP3: NOD-like receptor protein 3; LCMV: Lymphocytic choriomeningitis virus; ROS: Reactive oxygen species; RSV: Respiratory syncytial virus; SARS-CoV-1 and 2: Severe acute respiratory syndrome coronavirus 1 and 2; TGF-beta: Transforming growth factor beta; TNF-α: Tumor necrosis factor-alpha.

## 2. Overview of the First Responders to Viral Infection and Their Impact on the Outcome

All viruses are obligate intracellular parasites that require the host for their survival and replication. Vertebrate hosts try to keep themselves free of such invasions and have a wide range of strategies to accomplish this task. The first responders are cells that recognize molecular patterns on viruses (pathogen-associated molecular patterns-PAMPs), and these are usually shared by many microbial invaders. There are several types of pattern recognition receptors (PRR) (see Table 2), and when these are triggered, the responder cells undergo several molecular changes that enhance their properties and facilitate viral control. Prominent among these activities are the generation and release of molecules that are inhibitory to viral survival and replication. These include at least three different types of interferons (type I interferons include IFN-alpha and IFN-beta, type II interferon, also termed IFN-gamma, and type III interferon or interferon lambda) that exhibit antiviral activity in various ways. Additional molecular changes result in the generation of chemical mediators that recruit and activate cells that participate in an inflammatory reaction. This response generates activities that can inactivate invaders and suppress the infection. In the meantime, the virus or its components are taken up by cells, primarily dendritic cells, and presented to lymphoid cells that respond specifically to viral molecules. The lymphoid cells expand their numbers and functions, and some generate specific molecules (antibodies) that bind to the viral components. These types of responses serve to stop the establishment of a virus invader unless the virus has properties that can blunt or bypass the activity of one or more innate recognition systems or adaptive immune effectors that are generated. The latter type of viral agent is the topic of this review. Such viruses all possess properties that either manage and skirt effective innate responses or resist control by the nonlymphoid and lymphoid components of the inflammatory reaction. This begs the question of whether there are any practical ways to manipulate one or more aspects of innate immunity that will succeed in minimizing or preventing tissue-damaging viral infections. In a subsequent section, we focus on the role of adaptive immune responses in chronic reactions and ways to manipulate these aspects to minimize tissue-damaging lesions.

## 3. The Principal Components of Innate Immunity That Affect the Outcome of Viral Infections

**Figure 1:**
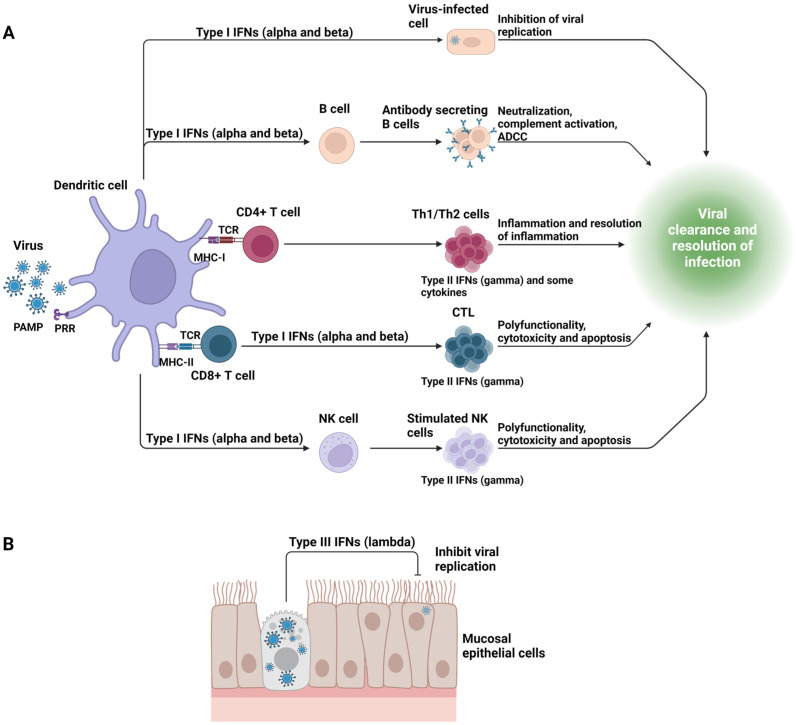
Some functions and activation of three types of interferons in response to viral infection. (**A**) shows that upon recognition of viral Pathogen-Associated Molecular Patterns (PAMPs) via pattern recognition receptors (PRR), dendritic cells become activated and secrete Type I Interferons (IFN-alpha and -beta). These Type I IFNs play a pivotal role in inhibiting virus replication in the infected cells and initiating the adaptive immune response. Subsequently, adaptive immune cells, including CD4+ T cells, CD8+ T cells, and specialized Natural Killer (NK) cells, respond to the Type I IFNs and T cell Receptor (TCR) signals by secreting Type II Interferons (IFN-gamma). This cascade of events enhances the function of adaptive immune cells along with NK cells and stimulates B cells to produce antibodies that neutralize viruses, leading to the clearance of infected cells. (**B**) shows that following infection of epithelial barrier cells by viruses such as measles, mumps, respiratory syncytial virus, or influenza, these cells express Type III Interferons (IFN-lambda). Type III IFNs act locally in a paracrine manner to control or inhibit viral replication within the infected cells, thereby helping to limit the spread of an infection (MHC-I/II: Major Histocompatibility Complex Class I/II; CTL: Cytotoxic T Cell. Figure created with BioRender.com; access date 27 March 2024).

The innate immune system is carried out by multiple cell types and several proteins of which the most relevant for virus infections are the interferons. Characteristically, innate defenders are ready for prompt action; they show no or limited selectivity and respond in a similar way when re-exposed to the same virus infection. Of the cellular components, the heterologous population of dendritic cells (DC) is an early participant [32]. These cells respond to viral PAMPs and produce cytokines that can be relevant for viral control, such as interferons, chemokines that attract other cell types, and cytokines involved in the induction of adaptive immunity. Some subsets of DC process viral antigens are involved in inducing specific antibodies and T cell responses (Figure 1A). Multiple experiments using model systems have documented how expanding, activating, or ablating one or another subset of DC impacts the pattern of antiviral immunity [32]. For example, several adjuvants that target DC are used to enhance antiviral immunity [33], but targeting DC to prevent damaging lesions in clinical situations is not yet a practical procedure. 

Other prominent innate cell types that respond to viral infections include macrophages, natural killer (NK) cells, and neutrophils. An abundance of investigations has focused on NK cells that can play a critical role in antiviral immunity [34]. Thus, without NK cells, animals become more susceptible to several viral infections [34,35,36]. NK cells also show a modicum of immunological memory and perform more effectively when re-exposed to the same virus infection, as has been well documented with cytomegalovirus infections [37]. NK cells function by causing apoptosis of infected cells and produce cytokines involved in antiviral defense. However, modulating NK cell numbers and functions is not currently a practical approach to shaping the outcome of a natural viral infection. Neutrophils, and to a greater extent macrophages, are other innate cell types that respond to viral infections. Both participate in early responses to infection, but both, particularly macrophages, may be more relevant in shaping the outcome of an established infection, especially those that become chronic. Accordingly, activated macrophages play a major role in causing tissue damage, especially a subtype of such cells referred to as M1 macrophages [38]. Several studies with model systems have shown that removing macrophages or changing the response to favor M2 over M1 dominance [39,40] serves to diminish inflammatory lesions, as is further discussed in a later section.

There are additional cell types that can contribute to innate immunity to viral infections. These include innate lymphoid cells (ILC) of different types and gamma delta T cells. The ILC lacks antigen-specific receptors and canonical markers of several better-investigated cells of the immune system. The ILCs themselves fall into at least three subtypes based on their major location, their expression of transcription factors, and the cytokines and chemokines they can produce [41]. The ILCs are primarily situated at barrier surfaces, especially mucosae, and are assumed to help protect these locations during primary infections. ILC is advocated to influence the outcome of some virus infections, such as the extent of liver pathology in hepatitis B infection [42] and possibly airway damage during influenza virus infection [43]. There is also some evidence that some ILCs may play a role in the repair of tissue damage via their ability to produce amphiregulin [43]. We cannot discount ILC as candidate cells to target to achieve a rebalanced immune response, but more information is needed before the approach can be used in clinical situations.

Similar caution may be merited with regard to targeting another less studied member of the innate immune fraternity, gamma/delta T cells. These cells do have T cell receptors and, in model systems, were shown to respond by producing inflammatory cytokines to several viral infections [44]. There are claims also that gamma/delta T cells can influence susceptibility to some viral infections [45] and that modulating their activity, as can be achieved by targeting the mevalonate pathway, can achieve less viral immunopathology in model systems [45,46]. More studies are needed to fully assess the role of gamma/delta T cells in chronic viral infections in human diseases.

Several host proteins already present in the body or released from innate cells that respond to viruses can shape the outcome of infection. With viruses, the most prominent are three classes of proteins called interferons (see Figure 1). The most relevant early responder interferons are type I, of which there are two subtypes, alpha and beta, and type III or lambda interferon [47]. Interferon type I alpha is present in large amounts in plasmacytoid DC, and this is rapidly released when such cells are exposed to a virus expressing a PAMP [48]. Interferons act in a paracrine fashion and bind to specific receptors on cells, usually those infected by the virus, and induce multiple changes in gene expression that are referred to as interferon response genes (ISGs). The ISGs mediate a wide range of biological responses, including the development of an antiviral state that involves multiple molecular events [49]. Type I interferons (IFNs) also impart immunomodulatory effects on other immune cells. For instance, NK cells responding to type I IFNs undergo changes such as increased antiviral potency by 10–100-fold [50]. Type I IFNs also recruit innate cells, enhance the activity of DCs, and promote adaptive immune responses [51].

Type III interferons (IFN-λ), of which there are four types, are also induced rapidly after virus infections, but they act on a narrower spectrum of cell types, which serves to limit the unwanted systemic inflammatory effects typical of interferon type I. The antiviral effects of IFN-λ are focused on epithelial and barrier surfaces, and IFN-λ may be more relevant than other interferons to protect against epithelial invasion by viruses [52]. In this context, it was noted that nasal epithelial cell responses to mumps, measles, and RSV are dominated by IFN-λ but not type I IFNs [53]. Moreover, IFN-λ was shown to control respiratory viral infections such as influenza virus infection and RSV infection (Figure 1B) [54,55]. Recently, IFN-λ, but not type I interferons, were shown to efficiently control rotavirus infection in human intestinal epithelial cells, indicating a division of labor among type I and type III interferons [56]. In conclusion, it could be that therapy with IFN-λ might be more effective than other interferons to protect barrier sites during initial infection, but this issue is of less relevance to shaping the outcome of established viral immunological lesions.

Type II interferons are mainly products of the adaptive immune system and function to participate in inflammatory reactions to viral infections. Manipulating type II interferon responses to control the expression of viral infections has mainly been investigated in model systems.

Meanwhile, interferons may play an active role in antiviral immunity, but if the response is not appropriately regulated, then untoward effects may occur. Thus, overproduction of type I IFN can interfere with effective immunity to SARS-CoV-2 infection, leading to more severe clinical consequences [57,58]. In well-studied model systems, persistent activation of IFN signaling is associated with hyperimmune activation and disease development in the context of some chronic infections. For example, two independent reports demonstrated that blocking type I interferon signaling in mice led to a favorable outcome during chronic LCMV infection, and this protective effect was dependent on CD4+ T cells [59,60]. Furthermore, it was shown that blockade of IFN-β 1 day prior to infection led to better control of chronic LCMV infection in mice [61]. The tissue-damaging effects of IFNs are not limited to chronic virus infections since, in severe acute influenza, increased levels of IFN-α/IFN-β may contribute to immunopathology [62].

Overall, these findings indicate that rebalancing innate immune aspects such as IFN-β and IFN-λ signaling represents a therapeutic approach to control chronic virus infections, but there is a delicate balance between achieving favorable rather than beneficial effects. Thus, further investigations are needed before clinically useful ways are developed to diminish viral immunopathology.

## 4. Targeting Innate Immune Components to Minimize Pathology Associated with Viral Infections

As discussed previously, innate immune components react to viruses as first responders and also as effectors in tissue damage. By far, the majority of experimental studies that assess the relevance of innate immunity during viral infections make changes before or early after virus infection. These investigations have provided valuable insight into how various innate components can act to control infections, but from a clinical perspective, we are usually faced with the need to suppress the impact of already established chronic infections. An abundance of investigations has shown that changing innate immune responsiveness prior to or early during infection can markedly affect the outcome. Several approaches have been used (see Table 3), and these results can show how different cellular and chemical mediators of innate defenses impact the outcome of a virus infection.

Several aspects of innate immunity contribute to tissue damage, and therapies that diminish such activities represent a valuable therapeutic objective. For example, experimental studies with several inflammatory viral infections have shown that destroying macrophages, as can be achieved by administering clodronate liposomes, which are taken up by phagocytic cells, alleviates lesion severity [88]. However, to our knowledge, this approach has not been used to control viral inflammatory lesions in natural disease situations. With regard to the pathological role of macrophages, it has been well established from model studies that tissue damage is usually associated with a subset termed M1, with another subset, M2, being relevant for the resolution of tissue damage [39,89]. Accordingly, changing the induction scenario to suppress M1 and/or expand M2 can, in model systems, result in diminished viral immunopathology [39]. There are also reports that suppressing M1 macrophage activity in established lesions can be beneficial [38,72], but such reports have yet to be translated for use in the clinic.

Perhaps the most effective approach that targets innate immune events to control viral inflammatory lesions has been to use specific moAbs to counteract some of the inflammatory molecules produced mainly by innate cells (Table 3). This approach has proven valuable in treating severe COVID lesions, but usually, in affected persons, additional therapies are also administered, such as anti-inflammatory drugs and perhaps antivirals, so assessing the true value of the moAbs is problematic. In the case where persons develop severe inflammatory reactions in Dengue hemorrhagic fever, anti-cytokines were shown to be useful in counteracting the so-called cytokine storm [72]. A notable disadvantage of using anti-cytokine therapy is its high cost, some side effects such as allergic reactions, and the increased likelihood of flare-ups of other chronic infections such as TB and conceivably some unrevealed cancers.

We must conclude that reshaping innate immune responsiveness to counteract the likely development of chronic inflammatory lesions to virus infection is a potentially useful strategy, but there are few if any, opportunities to use it in a practical clinical situation. Counteracting and even reversing established lesions by changing innate immune functions provides another opportunity for therapy that has much support from studies of model systems. This approach has much support from studies of model systems but little, if any, in clinical situations. What appears most promising in controlling clinical situations has been the administration of moAb to counteract inflammatory mediators. We anticipate that ongoing research will reveal valuable additional strategies.

## 5. Overview of the Principal Adaptive Immune Components That Participate in Viral Immunopathology

The idea that a reaction by a normally functioning immune system was responsible for the lesions observed following a virus infection first emerged from studies in mice with the non-cytopathic virus Lymphocytic choriomeningitis virus (LCMV). As its name suggests, this virus can cause choriomeningitis, which usually requires that the virus-infecting strain is delivered into the cerebrospinal fluid space. The inflammatory response that followed contains mainly lymphocytes. Moreover, as early studies by Rowe and colleagues showed, this reaction did not occur, and animals survived if mice were irradiated [90] or thymectomized prior to infection [91]. Other groups showed reactions did not occur if infected mice were immunosuppressed in various ways or genetically unable to mount immune responses [92]. This raised the idea that the immune response to the infection and not the virus itself accounted for the lesions. Subsequent studies by many groups, particularly those led by Oldstone, Zinkernagel, and Blanden, assembled a wealth of data showing that lesions in the brain, liver, and other sites resulting from LCMV infection represented reactions involving virus-specific T cell responses and that the cells were principally CD8+ T cells [93,94]. In other infection circumstances, these same CD8+ T cells can play an immune protection function against LCMV [95]. As the late Michael Oldstone liked to point out, studies using the LCMV model of infection have revealed a large fraction of our basic understanding of viral immunology and pathogenesis. Indeed, many ‘firsts’ came from LCMV investigations and some of the additional mechanisms discovered applied to immunopathology. An early mechanism discovered with LCMV was the observation that tissue damaging lesions resulting from a virus infection could also be caused by immune complexes composed of viral components bound to specific antibodies, with the complexes activating the complement system and generating an inflammatory reaction at the site they became entrapped [95]. These locations included the glomeruli of the kidney, the choroid plexus and site in the skin. Some additional examples of immune complex lesions during other viral diseases are mentioned in Table 1.

In the case of most pathologies that occur during LCMV, the cells orchestrating lesions are CD8+ T cells, with CD4+ playing far less or no role. CD8+ T cells also may be the major subset involved in some other viral immunopathologies, such as infections caused by HBV, which is a noncytopathic virus and perhaps in some aspects of human immunodeficiency virus (HIV) infection [96,97]. Many studies have been performed to define how the CD8+ T cells mediate tissue damage and to answer questions about the antigen specificity of the reaction. These studies showed that the majority of CD8+ T cell orchestrators were antigen-specific, but some recruitment of lymphoid and especially nonlymphoid cells into lesions also occurred. There is strong evidence that direct killing of infected cells is an effective mechanism, and CD8+ T cells genetically unable to kill, do not cause lesions [98]. There is also evidence that inflammatory cytokines are involved in tissue damage, a mechanism more common for CD4+ T cell reactions, as described subsequently [99,100]. One study maintained that CD8+ T cells, upon binding to infected targets, could purge cells of some components, which could conceivably involve some so-called luxury functions such as hormone production [101]. The protective and inflammatory consequences of immune CD8+ T cells reacting with antigen-expressing targets are not always fulfilled. Thus, as also discovered with LCMV, under conditions of high antigen load, the T cells may become exhausted and malfunction, as first described by the Zinkernagel group [102]. Much is now known about the mechanisms that explain immune exhaustion during chronic viral infections. These include the important discovery that reversing immune exhaustion using antibodies that block the effect, so-called checkpoint inhibitor therapy can result in the more effective control of chronic viral infections [103].

Although investigations using LCMV laid the groundwork for much of our understanding of viral immunology, the notion that CD8+ T cells seem to do everything could be misleading. In natural viral immunopathological scenarios, many additional immune events are ongoing simultaneously, and some of these involve subsets of CD4+ T cells that recognize viral components in a different way than CD8+ T cells. There are a number of functionally different subsets of CD4+ T cells, all of which recognize viral-derived viral peptides bound to MHC class 2 proteins. The CD4+ subsets usually do not act directly by cytotoxic effects on infected targets but instead upon recognizing antigen function indirectly by releasing cytokines and chemokines that are involved in recruiting additional cell types to the reaction, with these recruits mainly responsible for the tissue damage [96]. It is relevant to note that the CD4+ T cell response to a viral infection, as with the CD8+ T responses, mostly serves a protective role and usually contributes to controlling the infection. It is only when the response fails to achieve prompt control, and the virus is able to persist for a variety of reasons that the lesions then become chronic, and the reaction causes more tissue damage than occurs during a protective T cell-orchestrated inflammatory reaction. Several different subsets of CD4+ T cells can participate in viral immunopathology. The effector subsets are distinguished based on the types of transcription factors they produce, the chemokine receptors they express, and the effector molecules they produce when activated following antigen recognition [104]. In viral-induced immunopathology, the two subsets that mainly participate are termed Th1 and Th17 cells, and a third, called Th2, is associated with some cases that include lung lesions caused by RSV infection [105]. In most instances of viral immunopathology, Th1 cells are the predominant effectors, especially in early lesions, as our group described in a model of HSV-induced ocular lesions [11]. Subsequently, Th17 cells may take over as the main orchestrators of chronic inflammation.

Infection with HSV is highly cytolytic and induces a prompt reaction that usually succeeds in controlling the infection, and a long-lasting immune response is induced that largely protects against reinfection [106]. However, an invariable consequence of infection is that the virus seeds into the local peripheral nerve ganglion, where it sets up an alternate lifestyle in some neurons, which is referred to as latency. This is usually maintained indefinitely in the host, causing no obvious consequences. In its natural human host, the latent infection in some neurons breaks down, and the virus reinvades surface sites where it may cause a recurrent lesion. If these reactivation events occur in the eye, the reaction, particularly after several such episodes, can result in chronic inflammation and scarring that impairs vision [107]. This herpetic stromal keratitis (HSK) reaction is considered to be immunopathology involving T cells and is controlled in humans with anti-inflammatory drugs along with antivirals [108]. A similar immunopathological reaction occurs in mice following primary ocular infection with HSV, and this model has been used to identify the several steps involved in pathogenesis [109,110]. Early innate immune events are induced by viral replication that includes neutrophil and NK cell invasion as well as neovascularization of the normally avascular cornea [109]. The initial response may recede, and the virus largely controlled, but once the adaptive response is induced, T cells that in most models are mainly CD4+ T cells and additional nonlymphoid inflammatory cells invade, neovascularization is increased, and the reaction becomes chronic, usually failing to resolve [111]. In the early stages, Th1 cells predominate, but in later stages, Th17 cells may become more numerous than Th1 cells. This model has proven useful to verify the immunopathological nature of HSK and to find novel ways of diminishing its severity. For example, it was the model used that first documented a role for regulatory T cells (Treg) to control the extent of viral inflammatory reactions [112,113], the relevance of angiogenesis for corneal pathology [114] and the value of therapeutic procedures such manipulating microRNAs [115], epigenetic regulation [116] and the value of changing metabolic environments [117] to control immunopathology. These issues have been reviewed in more detail elsewhere [71]. 

Another natural example of CD4+ T cell-mediated immunopathology occurs with COVID-19 infection, although in this case, additional pathogenic mechanisms are likely contributing, but much remains uncertain since longitudinal invasive studies are not possible with infected humans and limited animal model systems are available for detailed study [118,119]. The immunopathological phase of COVID-19 infection does not occur in all SARS-CoV-2 infected patients. It does occur when the virus is not fully controlled in the upper respiratory tract but instead spreads to infect alveolar cells in the lower lungs. This outcome may be influenced by the effectiveness of the type one interferon response made initially with SARS-CoV-2, which is able to impair this response [120]. The inflammatory reaction contains many Th1 T cells, and the amount of interferon-gamma they produce may be a critical determinant of the outcome [121]. Virus replication may be largely controlled, but the inflammatory reaction may increase in magnitude and include multiple cell types such as macrophages, neutrophils, NK cells, and lymphoid cells. The reaction may progress in severity, with lung function becoming markedly damaged, the patient requiring ICU attention, and death a common outcome. It remains unclear as to the factors that affect the variable outcome, but a major event occurring is a cytokine storm with the inflammatory molecules deriving from macrophages, neutrophils, and other cell types that could include several subsets of T cells [1,2]. It is likely that the extent of CD4+ and CD8+ T cell involvement is a relevant issue, with these T cell subsets contributing to immunopathology and eventually orchestrating its control. It is evident that in severe cases, Th17 cells become prominent, and these attract and activate neutrophils, causing further inflammation [122]. Control measures that work most effectively against the reaction include anti-inflammatory drugs and moAbs that target cytokines such as IL-6, IL-1β, TNF-α, IL-4, IL-13, and IL-17A and chemokines such as CXCL-8, CXCL-10, and CCL2 may be very effective [123]. Inflammatory reactions to SARS-CoV-2 infection may involve additional organs, including the myocardium, kidneys, and liver. An additional complication with COVID-19 is that late-developing lesions can occur in some persons that collectively are referred to as long COVID. The mechanisms involved in long COVID are thought to be multiple, including the induction of autoinflammatory lesions [124,125]. 

As mentioned above, there is one example of a viral immunoinflammatory lesion that occurs with RSV that is orchestrated primarily by CD4+ Th2 cells [126,127]. These types of lesions are more characteristic of inflammatory reactions to parasites and to allergens. In fact, RSV infections in children are thought to be a risk factor for developing subsequent problems with asthma and allergies [128]. The principal cytokines produced by Th2 are IL-4, IL-5, and IL-13, as well as the chemokines that attract eosinophils and basophils. Thus, the makeup of these reactions differs from those orchestrated by Th1 and Th17 cells, and the lesions generate a notable quantity of secretions, which impedes air intake in infants. The Th2 cells act to recruit eosinophils and basophils to the lungs, involved in an IgE response, and this establishes an aggressive inflammatory hyperresponsiveness in the respiratory tract without efficient clearance of RSV [129,130]. Fortunately, RSV is becoming a less troublesome viral pathogen because an effective vaccine for adults and pregnant mothers was recently developed. In addition, a superior moAb is now available to protect infants from severe disease [131,132]. However, we still await a safe vaccine that can be used to protect young children, the most relevant sufferers of RSV infection [133].

Whereas inflammatory responses to viruses can involve several antigen-specific and recruited non-specific cell types included in the reaction, there are other cells and soluble mediators that play a counter-inflammatory function. Prominent among the cells involved in this function are the so-called Treg. Multiple cells can have a regulatory function, but the most studied cell type has been a subset of CD4+ T cells that express the high-affinity receptor for IL-2 and the transcription factor FoxP3 [134]. Another well-studied cell type is CD4+ T cells, which produce an abundance of the anti-inflammatory cytokine IL-10 [135]. As has been shown by many experimental models and also to a lesser extent during in vivo reactions to viruses, if functional Treg is absent or deficient, the inflammatory responses in chronic infections are more severe but can be limited in extent when Treg numbers are increased by some procedure [136]. Thus, a potentially valuable procedure to manage the severity of inflammatory reactions to viruses is to manipulate the involvement of Treg of various types or the cytokines they employ to mediate their activity. This topic has been discussed more thoroughly in previous reviews by our group and others [134,137].

## 6. Some Approaches Available to Diminish the Impact Lesions Caused by Adaptive Immune Responses to Viruses

It is customary to control the impact of viral immunopathological lesions using anti-inflammatory drugs such as corticosteroids, but the use of such drugs, particularly long-term, can have many undesirable side effects. However, as we mentioned previously, during immunopathological responses to viral infections, only some aspects of immunity contribute to tissue damage. At the same time, other immune components are also reacting, and these may have counter-inflammatory effects. Thus, an overall strategy to control the reaction could be to find ways to rebalance the involvement of different components of immune reactivity. We developed this idea and described in detail the many approaches that could be used to rebalance immune reactivity in a recently published review [71]. The numerous approaches explored and found to be effective were performed predominantly using model systems and used immune modulators either before or early after viral infection. Examples of success are listed in Table 4. In the clinic, the usual challenge is to reduce or even eliminate ongoing viral immunoinflammatory lesions. In this article, we briefly describe some approaches that could be the most practical to explore in clinical situations.

The recent occurrence of the COVID-19 pandemic was a boon to experimental pathologists and drug developers. Thus, as described in a previous section, many of the more damaging lesions that occur in patients infected with SARS-CoV-2 are immunopathological, and these can be lethal. Whereas antivirals used to treat such patients were usually ineffective, corticosteroid therapy was often efficacious. There were also successes recorded for using moAb to counteract cytokines and chemokines, which are products of both lymphoid and nonlymphoid inflammatory cells [152]. For example, IL-6 inhibitor therapy with tocilizumab and sarilumab is being evaluated as a potential treatment for COVID-19. Both drugs have shown effectiveness in individuals infected with COVID-19, highlighting the crucial therapeutic role of IL-6 blockade [152,153].

Other therapies explored included drugs that selectively disarmed the metabolic activity of proinflammatory T cells, such as mTOR inhibitors, metformin, statin, and 2-deoxy-glucose [154,155,156,157]. Isolated reports describe success with these therapies, although the data are invariably unconfirmed. Additional strategies worth pursuing could be the use of drugs such as gemfibrozil that modulate the nuclear receptor peroxisome proliferator-activated receptor (PPAR), which is involved in modulating glucose and lipid metabolism as well as the expression of some genes involved in inflammation [158]. A study using gemfibrozil showed an increased survival rate from 26% to 50% in influenza-infected mice when treatment started 4 days after infection. This effect is possibly due to the activation of the anti-inflammatory IL-4 cytokine production and a decrease in the inflammatory immune response [145].

If the window of opportunity for therapy is wider, then approaches such as diet manipulation can result in immune rebalancing. Accordingly, manipulating the diet, preferably before or very early after a viral infection, can change the severity of inflammatory lesions caused by a viral infection [148]. Thus, in our own studies, we showed that increasing the dietary content of short-chain fatty acids such as propionate and butyrate before or at the time of infection or using an inhibitor of glutamine metabolism in the early phases of infection resulted in significantly reduced ocular inflammatory lesions caused by HSV infection [146,159]. Dietary manipulation can have effects on the gut microbiome and set the stage for the reduction in the production of Th17 T cells that are involved in many inflammatory reactions [160].

An approach to achieve immune rebalancing that functions well in an experimental setting is to use a range of manipulations that expand the population of cells and their products that exert regulatory functions and serve to lessen the impact of proinflammatory cell types. Several strategies achieve this objective, as has been extensively reviewed, but the few maneuvers that have been explored so far in the clinic have been directed at the control of autoimmune lesions, many of which involve similar mechanisms as occur in viral immunopathologies [161]. We feel that manipulating the activity of regulatory cells and their products has great promise, but currently, it is not practical to combat the impact of chronic viral infections.

One well-explored approach that achieves immune rebalance that is used effectively in the clinic, although rarely so far to counteract viral inflammatory lesions, is to use moAbs that bind to and counteract the function of inhibitory molecules such as PD-1, PD-L1, CTLA-4 to cause effector T cells to lose their protective function. Such inhibitors become dominant in circumstances where the effectors are overexposed to antigens, as happens in many cancers and some chronic viral infections, as was discovered initially in chronic LCMV infection [102]. It was shown that these so-called exhausted cells could be restored to functionality using moAbs that blocked the inhibitor effect [103]. The use of such checkpoint control inhibitor therapy is used to treat some cancers and was shown to be effective in initial trials to limit the severity of some chronic viral infections [162,163]. It seems likely that checkpoint inhibitor therapy will find increasing use in the future to counteract troublesome viral immunopathologies.

Finally, we anticipate that some drugs now widely used to control other chronic problems such as diabetes and obesity may show cross-over value in treating some chronic viral lesions. One such example is using glucagon-like peptide I receptor agonists, such as semaglutide, that have already been evaluated to suppress inflammatory lesions in COVID-19 patients with diabetes [164]. The drugs appear to act by suppressing proinflammatory signals such as NF-κB and TNF-α in inflammatory cells and also reduce the inflammatory mediator C-reactive protein that, in turn, stimulates the cAMP-PK pathway, preventing cell damage by reducing reactive oxygen radicals [165]. Some studies also show that glucagon-like peptide I receptor agonists can stimulate the expression of IL-10, which also has anti-inflammatory effects [166]. Since semaglutide and similar drugs are becoming widely used, it will be of interest to see what other chronic viral disease lesions will benefit from their use. Additionally, of interest would be to learn if persistent users of semaglutide, which is necessary to control obesity, experience milder reactions to chronic infections and autoimmunities.

## Figures and Tables

**Table 2:** Some pattern recognition receptors that sense viral infections.

PRR	Viral PAMPs	Viruses	Refs.
TLR2	Envelope proteins	HSV	[20]
TLR3	dsRNA	HSV, MCMV, Rotavirus, Poliovirus	[21,22]
TLR4	Fusion protein	RSV	[23]
TLR7/8	ssRNA	RNA viruses	[24]
TLR9	dsDNA	DNA viruses	[25]
MDA5/RIGI	RNA	RNA viruses	[26]
cGAS	cytosolic DNA	HSV, HIV-1	[27,28]
NALP3 inflammasome	RNA, ion channels	RNA viruses HSV	[29,30]
AIM2 inflammasome	cytosolic DNA	MCMV	[31]

AIM2: Interferon-inducible protein or absent in melanoma 2; cGAS: Cyclic GMP-AMP Synthase; dsDNA: Double-stranded DNA; HIV-1: Human Immunodeficiency Virus type 1; HSV: Herpes Simplex Virus; MCMV: Murine Cytomegalovirus: MDA5/RIGI: Melanoma differentiation-associated protein 5; Retinoic Acid-Inducible Gene I; NALP3: NACHT, Leucine-rich repeat, pyrin domain-containing protein 3; PRR: Pattern recognition receptor; RSV: Respiratory Syncytial Virus; ssRNA: Single-stranded RNA; TLR: Toll-like receptor.

**Table 3:** Approaches targeting the innate immune system to mitigate viral diseases.

Strategies	In Vivo phenotype	Refs.
Macrophage directed	(i) Depletion of macrophages using clodronate liposomes affected viral disease outcome	[63]
	(ii) Targeting proinflammatory macrophages and pyroptosis affected COVID-19 outcome in murine models	[64,65]
	(iii) Administration of drugs or select cytokines-induced anti-inflammatory M2 macrophages leading to attenuation of viral pathology	[39]
Neutrophil directed approaches	(i) Neutrophil depletion using moAb attenuated HSV-1 induced ocular lesions	[66,67]
	(ii) Disrupting neutrophil extracellular traps mitigated multiple organ injury in COVID-19 mouse model	[68]
Cytokine directed approaches	Blockade of IL-6, IL-1b mitigated HSK lesion severity	[69,70]
	Inhibition of IL-1, IL-6, IL-17 impacted COVID-19 disease	[71]
	Targeting of TNF-α attenuated dengue lesions	[72]
	Inhibition of IL-1β and TNF-α reduces influenza severity in mice	[73,74]
	Blockade of interferon beta-controlled chronic LCMV infection	[59]
	Interferon lambda administration controlled Zika virus in the female reproductive tract	[75]
Chemokine blockade	Blockade of CCR2 CXCR3 was effective to mitigate influenza lesions	[71]
	CCR5 inhibition conferred benefits in COVID-19 disease	[76]
	CCR5 blockade impacted CCR5 trophic HIV-1 levels in affected patients	[77]
	CCR5 blockade impacted dengue disease development	[78]
Targeting Toll-like receptors/cytosolic viral sensors	Provision of TLR-2, TLR-3, TLR-4 agonists affected influenza disease in mice	[79,80,81]
	TLR-5 agonist flagellin cured rotavirus infection in mice	[82]
	TLR-7 agonist for human warts induced by papillomavirus	[83]
	TLR-7 agonist mitigated HBV and HCV disease	[84,85,86]
	NLRP3 inhibition reduced COVID-19 disease severity in mice	[87]

CCR2: Chemokine receptor; COVID-19: Coronavirus Disease 2019; CXCR3: Chemokine receptor type 3; HBV: Hepatitis B Virus; HCV: Hepatitis C Virus; HSK: Herpes Simplex Keratitis; HSV-1: Herpes Simplex Virus 1; LCMV: Lymphocytic Choriomeningitis Virus; M2: Macrophage subtype with anti-inflammatory properties; NLRP3: Nod-like receptor protein 3; TNF-α: Tumor necrosis factor alpha; TLR: Toll-like receptor.

**Table 4:** Models and some approaches for rebalancing participation of immune components.

In Vivo Model Systems	Example Approach	Refs.
Removing or blocking the products of proinflammatory T cells	IL-17R KO mice in HSV infection	[138]
	IL-6 deficient mice infected with influenza	[139]
Expanding the numbers and functions of regulatory cells and cytokines	Adoptive transfer of Treg cells in HSV-infected SCID mice	[136]
	Immune suppressive function of IL-10 in RSV-infected mice	[140]
Restoring lost protective cell function	Targeting exhausted T cells in LCMV	[141]
	Blockade of PD-1 and PD-L1 interaction with moAb in mice with HBV persistence	[142]
Exploiting differences in metabolic requirements of inflammatory and immunoprotective responses	Targeting mTOR in LCMV	[143]
	Targeting glucose and fatty acid metabolism in HSV infection	[144]
	Activating PPAR-α with an agonist molecule in influenza-infected mice	[145]
Changing nutritional environment during infection	Supplementing diet with short-chain fatty acid in HSV	[146]
	Consumption of prebiotics in inflammatory bowel disease	[147]
	High fiber diet supplemented mice infected with influenza	[148]
	Supplementing diet with short-chain fatty acid in HSV	[146]
Changing the expression of host molecules that impact on adaptive cell activities such as micro RNAs	Blocking miR122 with antagomir in HCV	[149]
	Using miR-155 antagomirs in ocular HSV infection	[150]
Adoptive transfer of cells that counter inflammatory reactants	Adoptive transfer of virus-specific B cells in LCMV infected model	[151]

HBV: Hepatitis B Virus; HCV: Hepatitis C Virus; HSV: Herpes Simplex Virus; KO: Knockout; LCMV: Lymphocytic Choriomeningitis Virus; miR: MicroRNA; moAb: Monoclonal antibody; mTOR: Mammalian target of Rapamycin; PD-1: Programmed cell death protein 1; PD-L1: Programmed death-ligand 1; PPAR-α: Peroxisome proliferator-activated receptor alpha; RSV: Respiratory Syncytial Virus; SCID: Severe combined immunodeficiency; Treg: Regulatory T cells.
